# A Call to Clarify the Intensity and Classification of Standing Behavior

**DOI:** 10.3390/ijerph18168460

**Published:** 2021-08-10

**Authors:** Robert J. Kowalsky, Lee Stoner, Mark A. Faghy, Bethany Barone Gibbs

**Affiliations:** 1 Department of Health and Kinesiology, Texas A&M University-Kingsville, Kingsville, TX 78363, USA; 2 Department of Exercise and Sport Science, University of North Carolina at Chapel Hill, Chapel Hill, NC 27599, USA; stonerl@email.unc.edu; 3 Human Sciences Research Centre, University of Derby, Derby DE22 1GB, UK; m.faghy@derby.ac.uk; 4 Department of Health and Human Development, University of Pittsburgh, Pittsburgh, PA 15260, USA; bbarone@pitt.edu

**Keywords:** sedentary behavior, sit-stand, posture, METs, cardiometabolic, physical activity

## Abstract

Public health guidelines for physical activity now include recommendations to break up prolonged sitting with light-intensity activities. Concurrently, interventions to increase standing have emerged, especially within the workplace in the form of sit–stand or standing workstations. Moreover, in short-duration studies, breaking up prolonged sitting with standing has been associated improved cardiometabolic outcomes. Publicly available estimates of the intensity of standing range from 1.5 to 2.3 metabolic equivalents (METs), neatly classifying standing as a light-intensity activity (>1.5 to <3.0 METs). Further delineation between ‘active’ and ‘passive’ standing has been proposed, with corresponding METs of >2.0 METs and ≤2.0 METs, respectively. However, this study reviews data suggesting that some standing (e.g., while performing deskwork) is substantially below the minimum light intensity activity threshold of 1.5 METs. These data bring into question whether standing should be universally classified as a light-intensity behavior. The objectives of this study are to (i) highlight discrepancies in classifying standing behavior in the human movement spectrum continuum, and (ii) to propose a realignment of the ‘active’ vs. ‘passive’ standing threshold to match the light intensity threshold to help provide a clearer research framework and subsequent public health messaging for the expected health benefits from standing.

## 1. Introduction

Sedentary behavior is a novel risk factor for poor cardiometabolic outcomes [1,2,3]. The strong relationship between sedentary behavior and cardiometabolic outcomes was emphasized in the 2018 Physical Activity Guidelines Advisory Committee Report [4], the 2020 Canadian 24-h Movement Guidelines for Adults [5], and the World Health Organization’s Guidelines on Physical Activity and Sedentary Behavior [6]. These guidelines recommend substituting sitting with light-intensity physical activities, such as walking and, in the Canadian guidelines, standing. Considering this, standing is an attractive strategy for interrupting prolonged sitting (sitting accumulated in long periods of time, e.g., 1 hour), not least because it is relatively simple to implement in many environments where sitting is common, such as the workplace. Moreover, the use of sit–stand desks in laboratory and simulated workplace settings have demonstrated improved acute cardiometabolic outcomes [7,8]. These data suggest that standing could be an important tool in the fight against the public health problem of sedentary behavior, especially in the workplace. However, not all scientists acknowledge standing as an effective sedentary behavior replacement strategy [9], and a major argument against standing is its incremental contribution to caloric expenditure [10].

The Sedentary Behavior Research Network’s (SBRN) 2017 consensus definition of sedentary behavior has two components: (i) a seated/reclined/lying posture and (ii) a low intensity of ≤1.5 METs [11]. This consensus statement also defines standing as behavior in an upright posture and delineates two subcategories: ‘active standing’, which is >2 METs, and ‘passive standing’, which is ≤2 METs [11]. Both forms of standing can include supported or unsupported standing, as well as standing with shuffling and weight shifting, only delineated by the level of energy expenditure. Examples of active standing could include standing while doing dishes and standing while on an assembly line, with passive standing including examples such as standing in a line or having a discussion with an individual. Additionally, published estimates of the intensity of standing fall between 1.5 to 2.3 METs, neatly classifying standing as a light-intensity activity [12]. A limitation of this previous research, though, is that the intensities (MET values) of some activities are based on estimated values determined from similar activities instead of direct measurements. However, research that carefully measured energy expenditure during minimally active standing activities, such as deskwork [13,14], suggested that standing with limited other activity has an incremental additional expenditure that is typically below the 1.5 MET threshold of light intensity. This inconsistency brings into question whether standing should be universally classified as a light-intensity activity for the purposes of research and public health messaging.

## 2. Intensity of Standing Desk Work

**Figure 1:**
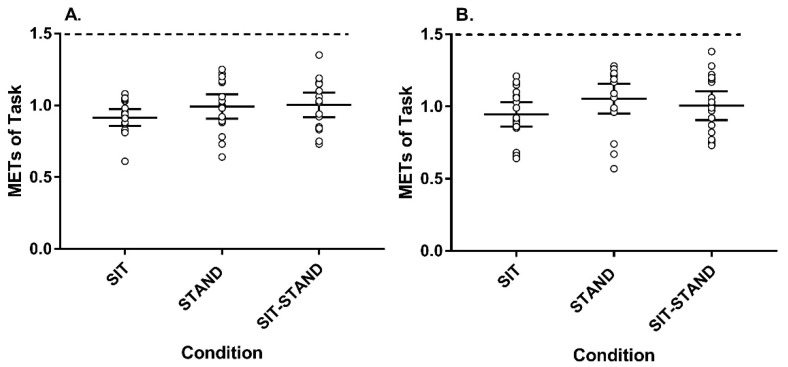
Intensity (METs) of typing (**A**) or writing (**B**) by condition. Horizontal lines indicate the condition mean and 95% CI, the dashed line represents the light intensity threshold. A (mean ± SD METs): SIT: 0.92 ± 0.16, STAND: 0.99 ± 0.20, SIT-STAND: 1.00 ± 0.24. B (mean ± SD METs): SIT: 0.95 ± 0.23, STAND: 1.06 ± 0.24, SIT-STAND: 1.01 ± 0.26. SIT: 60-min sitting condition, STAND: 60-min standing condition, SIT-STAND: 30 min of standing followed by 30 min of sitting condition.

To help bring further light to this issue, we present individual-level data from our previously published work that evaluated the energy expenditure of completing standardized deskwork in a seated, standing, or sit–stand posture (*n* = 18) [13]. This study reported that, compared with 1 h of sitting (SIT), standing for 1 h (STAND) or alternating sit–stand for 1 h (30 min of each; SIT-STAND) resulted in an additional 8.2 ± 15.9 kcal per hour and 5.5 ± 12.4 kcal per hour, respectively. Transforming these data to intensity levels (METs), Figure 1 displays the individual (open circles), group mean (longer horizontal bars), and 95% confidence intervals (shorter horizontal bars) for the intensity of each desk-based task (typing (A) and writing (B)) and by condition (SIT, STAND, and SIT-STAND). Using a one sample t-test for a comparison with the 1.5 METs threshold for light-intensity activity as the criterion, the average METs for each condition were significantly below the criterion, regardless of the task. For typing and writing, the average intensity during STAND was well below light intensity by −0.51 ± 0.17 METs (*p* < 0.001) and −0.44 ± 0.21 METs (*p* < 0.001), respectively. Further, no individual had >1.5 METs during any task or condition.

It should be noted that this protocol strived to maintain high internal validity by controlling participant pre-visit exposures (e.g., participants abstained from food for 8 h, as well as from moderate to vigorous intensity physical activity, alcohol, and nicotine for 24 h) and experimental conditions (temperature-controlled room, morning-only data collection, and confinement to the desk for the duration of the protocol). These protocol choices could have decreased the ecological validity of our finding and led to some underestimation of the intensity of the standing deskwork. Despite this, our estimates likely reflect energy expenditure while standing with limited movement in some situations, and metabolic accelerators such as the thermic effect of food and caffeine would be unlikely to increase the intensity of standing deskwork above the 1.5 METs light intensity threshold. This limitation is contrasted with the strengths of our study’s design, which included a representative sample that ranged in age (39 ± 13 years, range 22–57 years), represented both genders (*n* = 9 males; *n* = 9 females), and had variable body mass indices (25.6 ± 3.59 kg/m^2^, range: 17.5–30.6 kg/m^2^), along with the completion of ecologically valid tasks.

## 3. Discussion

**Figure 2:**
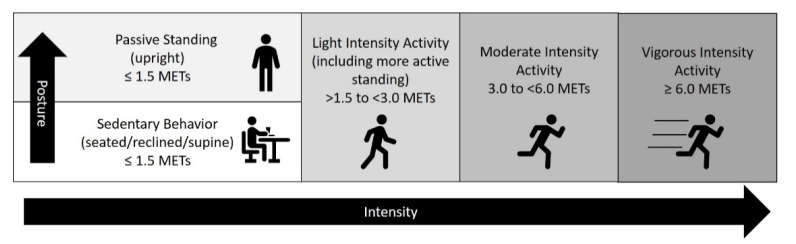
Proposed framework for classifying standing as part of the human movement spectrum.

Viewing these results in light of the definition of sedentary behavior provided by the SBRN [11], it appears that standing while completing typical deskwork in an occupational office setting may not meet the criteria for light-intensity activity or sedentary behavior due to its low intensity yet upright posture. The standing deskwork completed by the participants in our study could be accurately categorized as passive standing (<2 METs), yet was substantially below this threshold and straddled typically used categorizations of ‘sedentary’ and ‘light’ intensity. We support the SBRN’s helpful efforts to differentiate between types of standing (active vs. passive), yet, given that the intensity of standing behavior can fall below ‘light’, we propose a minor adjustment of the threshold separating passive from active standing to better align with established categorizations of activities across the human movement intensity spectrum. Our suggestion is that it is incorrect to classify all standing as light-intensity activity, just as all walking is not considered moderate-intensity activity [15]. Rather, an intuitive and harmonious framework could separate passive from active standing with alignment to the 1.5 METs threshold that also separates sedentary from light-intensity activity. This would result in any activity above the 1.5 MET threshold being classified as light-intensity activity, including active standing (Figure 2), and clearly separates activities below the 1.5 MET threshold by posture into sedentary behavior (seated/lying/reclining) and passive standing (upright).

Our proposed framework would provide clearer classifications to be used in research, particularly for the study of the health effects of increasing passive standing—an area in need of more research [5]. This improved clarity could also aid in the formation of public health messages regarding standing participation, for example, use of sit–stand desks. With current guidelines recommending limiting and interrupting sedentary behavior, it is important for scientists and public health messaging to specifically study and clearly describe the role of standing as a replacement behavior in multiple environments and forms. This must be undertaken with clear and consistent definitions and transparency in reporting; when possible, researchers should measure both posture and intensity in this pursuit.

A key implication of our findings, where passive standing has an intensity that is likely below the light intensity threshold, is that the cardiometabolic [7,8,16] and potential musculoskeletal benefits [17] from standing are likely not attributable to a meaningful increase in energy expenditure. Rather, these benefits derived from intermittent standing are likely multi-factorial and may be due to an attenuation of the effects of prolonged sitting, including negative cardiovascular and musculoskeletal effects [18,19,20,21] along with increased stress and forces on joints (e.g., lower back, neck) [22,23]. Future research delineating these mechanisms will be important for designing interventions and, eventually, clarifying public health recommendations with respect to passive standing in partnership with light and more intense physical activity recommendations.

## 4. Conclusions

In conclusion, we suggest that the intensity of standing with limited movement, e.g., completing desk-based tasks, is typically below the 1.5 MET threshold for light-intensity activity. As such, this passive form of standing cannot be classified as sedentary or light-intensity activity by current definitions. This discrepancy presents a research and public health challenge for how to study and recommend passive standing, a behavior with high feasibility and preliminary evidence of benefit. We propose delineating passive standing as upright behavior with an intensity of ≤1.5 METs and including active standing with an intensity of >1.5 METs in the category of light-intensity activity. We hypothesize that both standing and light physical activity have their place in the promotion of physical activity participation for health benefits, yet for different physiological reasons. Using this framework, we highlight that passive standing in particular is an area in need of further research regarding long-term health benefits. This research is important, as passive standing is a common strategy recommended and used to interrupt prolonged sitting. To further guide sedentary behavior-focused public health policy, the effects and feasibility of passive standing vs. light-intensity activities (including active standing) for interrupting prolonged sitting should be the focus of future studies aiming to design and recommend the most health-enhancing strategies for reducing sedentary behavior.

## Data Availability

The data presented in this study are available on request from the corresponding author.
